# Lifestyle, Inflammation, and Vascular Calcification in Kidney Transplant Recipients: Perspectives on Long-Term Outcomes

**DOI:** 10.3390/jcm9061911

**Published:** 2020-06-18

**Authors:** Camilo G. Sotomayor, Charlotte A. te Velde-Keyzer, Martin H. de Borst, Gerjan J. Navis, Stephan J.L. Bakker

**Affiliations:** Department of Internal Medicine, University Medical Center Groningen, University of Groningen, 9700 RB Groningen, The Netherlands; c.a.keyzer@umcg.nl (C.A.t.V.-K.); m.h.de.borst@umcg.nl (M.H.d.B.); g.j.navis@umcg.nl (G.J.N.); s.j.l.bakker@umcg.nl (S.J.L.B.)

**Keywords:** nephrology, kidney transplant, kidney transplant recipients, long-term outcomes, graft failure, cardiovascular mortality, lifestyle, inflammation, vascular calcification, bone mineral density, dual-energy X-ray absorptiometry

## Abstract

After decades of pioneering and improvement, kidney transplantation is now the renal replacement therapy of choice for most patients with end-stage kidney disease (ESKD). Where focus has traditionally been on surgical techniques and immunosuppressive treatment with prevention of rejection and infection in relation to short-term outcomes, nowadays, so many people are long-living with a transplanted kidney that lifestyle, including diet and exposure to toxic contaminants, also becomes of importance for the kidney transplantation field. Beyond hazards of immunological nature, a systematic assessment of potentially modifiable—yet rather overlooked—risk factors for late graft failure and excess cardiovascular risk may reveal novel targets for clinical intervention to optimize long-term health and downturn current rates of premature death of kidney transplant recipients (KTR). It should also be realized that while kidney transplantation aims to restore kidney function, it incompletely mitigates mechanisms of disease such as chronic low-grade inflammation with persistent redox imbalance and deregulated mineral and bone metabolism. While the vicious circle between inflammation and oxidative stress as common final pathway of a multitude of insults plays an established pathological role in native chronic kidney disease, its characterization post-kidney transplant remains less than satisfactory. Next to chronic inflammatory status, markedly accelerated vascular calcification persists after kidney transplantation and is likewise suggested a major independent mechanism, whose mitigation may counterbalance the excess risk of cardiovascular disease post-kidney transplant. Hereby, we first discuss modifiable dietary elements and toxic environmental contaminants that may explain increased risk of cardiovascular mortality and late graft failure in KTR. Next, we specify laboratory and clinical readouts, with a postulated role within persisting mechanisms of disease post-kidney transplantation (i.e., inflammation and redox imbalance and vascular calcification), as potential non-traditional risk factors for adverse long-term outcomes in KTR. Reflection on these current research opportunities is warranted among the research and clinical kidney transplantation community.

## 1. Introduction

Chronic kidney disease (CKD) is a major public health problem, with a current worldwide prevalence of approximately 843 million individuals [1]. Global mean prevalence was recently reported at 13.4% for all CKD stages together (1–5) and at 10.6% if only the more severe CKD stages (3–5) are considered [2]. Whereas the prevalence of all stages of CKD rises with age, older patients with similar levels of eGFR are less likely than their younger counterparts to progress to the need of renal replacement therapy, which has raised the question of whether all older patients who meet criteria for CKD actually have CKD [3].

**Figure 1:**
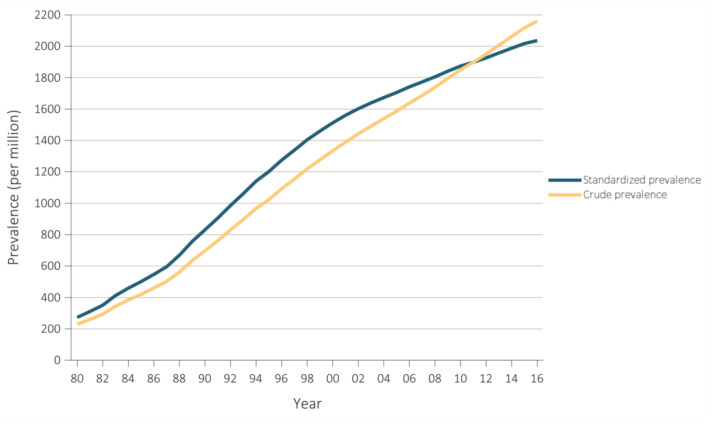
Prevalence of end-stage kidney disease (ESKD) in the United States (US) population, 1980–2016. This figure shows a steady increase in ESKD prevalence over recent ~35 years in the US. Standardized for age, sex, and race. Data Source: USRDS 2018 Annual Data Report [4].

**Figure 2:**
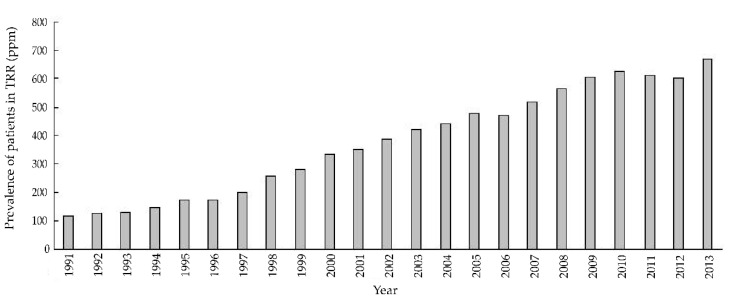
Prevalence of renal replacement therapy in Latin America, 1991−2013. This figure shows a steady increase in prevalence of renal replacement therapy over recent ~25 years in Latin America. Reprinted from “Latin American Dialysis and Transplant Registry: Experience and contributions to end-stage kidney disease epidemiology” [5].

The prevalence of CKD, its detection, treatment, and impact on health have been mainly studied in economically developed countries [1]. Nevertheless, even in these circumstances, it usually remains a silent, smoldering health threat, with, e.g., rates of awareness of being afflicted with kidney disease of approximately 10% among patients with CKD in an economically developed country like the United States [4]. Along the same line, in 2016, approximately 35% of patients diagnosed with incident end-stage kidney disease (ESKD) received little or no nephrology care prior to actually being diagnosed with ESKD [4]. Regrettably, prevalence of ESKD and prevalence of renal replacement therapy continue to increase (Figure 1 and Figure 2) [4].

Compared to chronic dialysis treatment, kidney transplantation is considered the renal replacement therapy of choice and the gold-standard treatment for most ESKD patients because it offers superior cost-effectiveness, quality of life, and life expectancy [6,7,8,9,10]. However, the latter has largely been due to significant improvements of short-term outcomes [11]. Advances in immunosuppression, tissue typing, treatment of infections, and surgical techniques led rates of 1-year graft survival at a pinnacle, whereas improvement of long-term outcomes post-transplant remains a major challenge in the kidney transplantation field [11].

On the one hand, the life-saving benefit of a kidney transplant remains largely hampered by cumulative injury of a multitude of hazards through immune and non-immune mechanisms of kidney damage. Over time, these mechanisms lead to chronic interstitial fibrosis and tubular atrophy as histopathological consequence and end-stage kidney allograft failure as functional repercussion, eventually requiring restart of dialysis or re-transplantation as final adverse clinical event (i.e., graft failure) [11,12,13,14,15].

On the other hand, kidney transplant recipients (KTR) are at particularly high risk of premature death, depicting overall mortality rates considerably higher than that of age-matched controls in the general population [16,17].

Indeed, approximately half of all kidney allograft losses are due to premature death with a functioning graft, a long-standing pattern that has remained largely unchanged over recent years [17,18].

**Figure 3:**
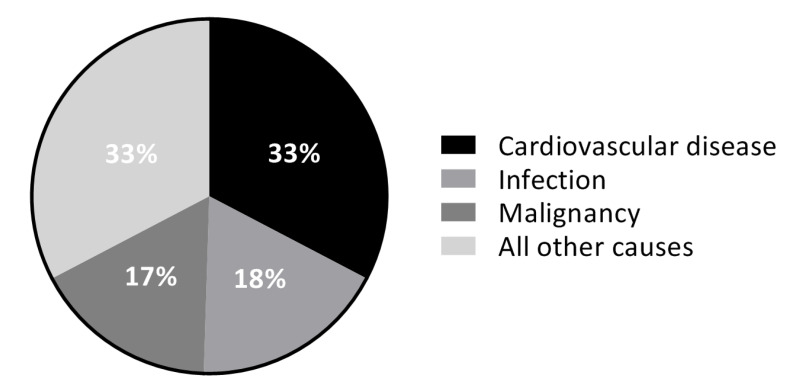
Mortality by causes of death with graft function in US KTR in 2015. This figure shows that cardiovascular disease was the leading cause of mortality among US KTR in 2015. Cardiovascular disease included acute myocardial infarction, atherosclerotic heart disease, congestive heart failure, cerebrovascular accident, and arrhythmia/cardiac arrest. Adapted from USRDS 2018 Annual Data Report [4].

Next, under the general understanding that cardiovascular disease is the leading cause of premature death post-kidney transplant (Figure 3) and thereby importantly challenging the improvement of longevity of KTR, great efforts have focused on the improvement of long-term cardiovascular outcomes [19,20,21].

In the clinical setting of KTR after the first-year post-transplant, beyond hazards of immunological nature, there is a pressing need to systematically study and characterize the clinical impact of potentially modifiable risk factors, such as lifestyle, diet, and exposure to toxic contaminants, which are underexplored areas in the kidney transplantation field [22,23,24,25,26]. This evidence is needed to guide decision making by clinicians and policy-makers in post-transplantation care. Furthermore, because kidney transplantation aims to restore kidney function but it incompletely mitigates collateral mechanisms of disease, such as chronic low-grade inflammation with persistent redox imbalance and deregulated mineral and bone metabolism, further research investigating specific clinical and laboratory readouts with a proposed involvement in such pathological pathways may point towards non-traditional risk factors and reveal novel targets for clinical intervention [27,28,29,30,31,32].

In the kidney transplantation field, future advances are expected from amelioration of adverse long-term outcomes by increasing recognition and developing novel, early, and cost-effective risk-management strategies focused on the non-immune aspects of post-kidney transplantation care and thus optimize long-term health and downturn current rates of premature death in stable KTR [11].

## 2. Lifestyle: Healthy Diet and Toxic Contaminants

One area with great potential for improvement is lifestyle, in particular diet and exposure to toxic contaminants. Systematic investigation of traditional and potentially modifiable risks factors in the post-kidney transplant setting may point towards otherwise overlooked early risk-management opportunities and thus provide the basis for the development of cost-effective interventional approaches to increase the lifespan of KTR. Healthy diet is a cornerstone element of cardio-metabolic health in the general population [33,34,35,36,37,38]. In general, a healthy diet is recommended as essential for cardiovascular disease prevention in all individuals. Surprisingly, however, little is known about the potential impact of a healthy diet on cardiovascular health and survival benefit in kidney patients across the continuum of CKD stages, in patients undergoing kidney replacement therapy, and remarkably limited evidence is available in the post-kidney transplantation clinical setting [39,40,41,42]. Moreover, native CKD and pre-transplant ESKD patients are generally advised to follow seemingly conflicting and challenging dietary recommendations with the aim of restricting individual nutrients such as potassium, salt, phosphorus, and protein [43]. It should be realized that there is scant evidence to support such restrictive dietary recommendations [44,45,46]. Finally, there is a notorious lack of studies aimed to aid on the development of evidence-based recommendations to appropriately adjust any pre-transplant dietary advice to the patient after kidney transplantation has been performed [26,43,44,47,48]. Below, we provide several examples of where opportunities may lie (Box 1).

Box 1 **Characteristics of a healthy diet [49].**

- ≥200 g of fruit per day (2–3 servings).
- ≥200 g of vegetables per day (2–3 servings).
- Fish 1–2 times per week, one of which to be oily fish.
- Saturated fatty acids to account for <10% of total energy intake through replacement by polyunsaturated fatty acids.
- Trans unsaturated fatty acids: as little as possible, preferably no intake from processed food and <1% of total energy intake from natural origin.
- 30 g unsalted nuts per day.
- <5 g of salt per day.
- Consumption of alcoholic beverages should be limited to 2 glasses per day (20 g/d of alcohol) for men and 1 glass per day (10 g/d of alcohol) for women.
- Sugar-sweetened soft drinks and alcoholic beverages consumption must be discouraged.

### 2.1. Fruit and Vegetable Consumption Post-Kidney Transplantation

With the aim of limiting potassium intake, for example, pre-transplant ESKD patients have largely been discouraged from a high consumption of fruits and vegetables, which are, however, well-known essential components of a healthy diet [50,51,52,53,54]. Beyond being rich in potassium, fruits and vegetables are rich in fibers, polyunsaturated and monounsaturated fatty acids, magnesium, iron, and generate less acid and contain smaller amounts of saturated fatty acids, protein, and absorbable phosphorus in comparison to meat [39,55]. At least four servings of fruit and vegetables per day are widely recommended for the prevention of major chronic diseases in the general population [49]. Indeed, increased consumption of fruits and vegetables has consistently shown to confer superior cardiovascular prognosis in the general population [52,53,54,56].

Recent studies show that KTR consume less fruits and vegetables than the general population, which has been associated with higher risk of cardiovascular mortality and posttransplant diabetes [57,58]. At present, however, post-kidney transplant, there is no clear incentive by transplant healthcare providers to prescribe restoration of the consumption of these basic items of a healthy diet. This attitude may respond to the fact that it remains relatively unexplored whether an increase of fruits and vegetables consumption post-kidney transplantation positively impacts outcomes of KTR, which would be hypothetically expected mainly by decreasing the excess cardiovascular burden and premature cardiovascular death. Epidemiological studies aimed to estimate a theoretical benefit of a relative increase of these specific food items are warranted as first step to, thereafter, investigate potential interventional strategies promoting novel, cost-effective, and patient-centered approaches to the nutritional management of KTR, adequately informing clinical practice and policy.

### 2.2. Fish Intake Post-Kidney Transplantation and Mercury Exposure

Similarly, fish are rich in the omega-3 polyunsaturated fatty acids (n-3 PUFA) EPA (eicosapentaenoic acid) and DHA (docosahexaenoic acid), which are suggested to yield several beneficial effects for cardiovascular health [59,60,61,62]. Circulating levels of EPA and DHA have been associated with reduced cardiovascular risk in both healthy populations and in patients with pre-existing cardiovascular disease [59,60,61,62]. Proposed beneficial health effects of marine-derived n-3 PUFA are wide-ranging, favorably impacting inflammation, fibrosis, lipid modulation, plaque stabilization, blood pressure, artery calcification processes, and endothelial function [63,64,65,66]. These properties render EPA and DHA as of encompassing therapeutic potential in the management of cardiovascular risk of KTR. Indeed, in this particular setting, recent observational studies showed that plasma levels of marine-derived n-3 PUFA are inversely associated with cardiovascular mortality risk [67,68].

It should be realized, however, that the results of randomized control trials using supplementation of these individual nutrients are not yet sufficiently powered to draw definitive conclusions and recommendations for KTR [69,70]. Moreover, no study has been devoted to evaluating the potential beneficial effect of a relatively high dietary fish intake, as mostly shown in the general population [71,72,73,74,75]. Indeed, fish is the main dietary source of n-3 PUFA, and its inclusion in diet seems reasonable because it is a good source of protein without potentially adverse effects of accompanying intake of high saturated fat as present in fatty meat products. Not exempt of drawbacks, however, fish is also the major source of human exposure to organic mercury (with the exception of industrial accidents or particular occupational exposures) [76,77,78]. Therefore, alongside the study of the potential health benefits of marine-derived n-3 PUFA, weighted investigation of a relatively higher fish intake has been performed as necessary step towards developing cautious evidence-based dietary guidelines for clinical uptake [79], suggesting that beneficial effects of a higher dietary intake of n-3 PUFA by increasing fish consumption post-kidney transplantation may not be mitigated by postulated increased cardiovascular risk due to concomitant exposure to mercury [79].

### 2.3. Cadmium Exposure and Nephrotoxicity in the Post-Kidney Transplant Setting

Cadmium is another heavy metal of environmental and lifestyle-related concern, with tobacco and diet as primary sources of exposure. Previous studies have demonstrated that cadmium may induce hypertension, which in turn is associated with accelerated kidney function decline and particularly demonstrated in KTR, by shortened allograft survival [80,81,82,83,84]. Most importantly, a strong body of evidence shows that the kidney is the most sensitive target organ of cadmium-induced body burden, through postulated direct mechanisms of cadmium-induced injury in this organ, wherein it accumulates with a half-life of up to 45 years [85,86,87,88,89]. It is important to note that, particularly in settings of long-term oxidative stress such as that of KTR, cadmium-induced nephrotoxicity may be associated with impaired kidney function at concentrations that are otherwise considered non-toxic [90,91,92]. Taking also into account that the most effective way to reduce cardiovascular disease in KTR may indeed be preservation of graft function, the aforementioned constellation of factors turn the investigation of cadmium-associated risk of encompassing relevance within the study of long-term outcomes of kidney allograft function [21,84]. Furthermore, bodily cadmium is susceptible to therapeutic interventions [93]. Thus, cadmium-targeted interventional strategies may offer novel opportunities to decrease the long-standing high burden of late kidney graft failure; however, whether the nephrotoxic exposure to cadmium represents an overlooked hazard for preserved graft functioning remains unknown.

## 3. Inflammation and Oxidative Stress and Vascular Calcification

Another area of great opportunities for further improvement may lie in a better evaluation of disease mechanisms long-term after transplantation. Traditional risk factors such as diabetes mellitus, smoking, and hypertension, among others, do not suffice to account for the excess burden of premature cardiovascular death of, otherwise, stable KTR [94,95,96,97]. Indeed, cardiovascular disease has an atypical nature in KTR when compared with the general population [20,21]. Unexplained cardiovascular risk subsidizes current efforts to provide cutting-edge evidence on the potential independent hazard of novel (non-traditional) cardiovascular risk factors post-kidney transplantation [98,99,100,101,102].

It should be taken into account that while kidney transplantation aims to restore kidney function, it incompletely abrogates mechanisms of disease. Moreover, an aggregate of factors specific to the transplant milieu such as a chronic low-grade immunologic response to the kidney allograft, long-term toxicity of maintenance immunosuppressive, as well as various degrees of progressive uremia, contribute to perpetuate chronic inflammation, redox imbalance, and deregulated mineral and bone metabolism, which have to be proposed as major independent and evolving pathophysiological mechanisms, whose mitigation may counterbalance—at least to a considerable extent—the excess risk of cardiovascular disease and graft failure post-kidney transplantation [30,32,101,103,104]. Below, we provide several examples of where opportunities may lie.

### 3.1. Inflammation and Oxidative Stress Post-Kidney Transplantation

Indeed, while the vicious circle between inflammation and oxidative stress as final common pathway of a multitude of insults plays an established pathological role in native chronic kidney disease (CKD), its characterization post-kidney transplant has been less than satisfactory [105,106,107,108,109]. This is relevant because, at a physiological level, the cornerstone role of the complex interplay between inflammation and oxidative stress (Box 2) provides a theoretical and conceptual framework upon which upcoming research may deepen the understanding of the pathophysiological status of KTR once they reach a seemingly stable clinical stage [105].

Box 2 **Oxidative stress.**

Oxidative stress is defined as an imbalance between the generation and removal of oxidant species. The most representative biological oxidant agents are reactive oxygen species (ROS) and reactive nitrogen species (RNS). The former group includes hydrogen peroxide, superoxide anion, and hydroxyl radical, whereas within the latter group relevant species are peroxynitrite anion, nitric oxide, and nitrogen dioxide radicals. Oxidative stress occurs when ROS and/or RNS production overwhelms the endogenous antioxidant defense system, either by excess production and/or inadequate removal. The antioxidant defense system is constituted by enzymatic antioxidant agents, including catalase, glutathione peroxidase, and superoxide dismutase. Non-enzymatic antioxidant components include a diversity of biological molecules, such as ascorbic acid (vitamin C), α-tocopherol (vitamin E), reduced glutathione, carotenoids, flavonoids, polyphenols, and several other exogenous antioxidants [110].

#### 3.1.1. Vitamin C as Anti-Inflammatory and Antioxidant Agent and Its Depletion Post-Kidney Transplant

Inflammation, specifically the established inflammatory biomarker high-sensitivity C-reactive protein (hs-CRP)—which is also an indirect marker of increased oxidant production—has been previously shown to be independently associated with increased mortality risk in KTR [98,100]. Supported by data consistently showing an inverse correlation with hs-CRP in different settings, vitamin C is well-known by its anti-inflammatory effects [111,112,113,114]. Moreover, vitamin C is a physiological antioxidant agent, with radical-scavenger and reducing activities, of paramount importance for protection against diseases and degenerative processes caused by oxidant stress [115]. This particular composite of biochemical properties renders vitamin C as compelling research candidate to broaden the understanding of the interaction of inflammation and oxidative stress in the mechanisms leading to excess risk of premature death post-kidney transplantation. It should be realized, moreover, that pre-transplant ESKD patients often have an imbalance of several critical trace elements and vitamins [39]. Vitamin C, particularly, has been shown to be removed by conventional hemodialysis membranes, leading to drastic vitamin C depletion and oxidative stress [116,117,118]. Through an inverse mediating effect on inflammatory signaling biomarkers, sub-physiological levels of vitamin C (depletion) may be hypothesized to be implicated in mechanisms that associate with increased risk of adverse long-term outcomes [119,120,121,122,123]. To date, however, relatively little is known regarding the prevalence of abnormal vitamin C status post-kidney transplantation, yet recent studies have shown that low plasma vitamin C contributes to excess risk for premature death post-kidney transplantation [124,125].

#### 3.1.2. Advanced Glycation End products as Amplifiers of Oxidative Stress and Inflammatory Responses

Inflammation is referred to as a redox-sensitive mechanism on the basis that reactive oxygen species may activate transcription factors such as nuclear factor kappa B (NF-kB), which regulates inflammatory mediator genes expression [126]. In this regard, advanced glycation end products (AGE) are particularly interesting oxidative stress biomarkers because it has been demonstrated that, upon binding to AGE-specific receptors, AGE activate intracellular pathways that amplify inflammatory and oxidative stress responses and regulate the transcription of adhesion molecules through NF-kB activation [127]. In agreement, data derived from clinical studies in pre-transplant ESKD patients support the implication of AGE in the complex feedback loop between oxidative stress and inflammation leading to endothelial dysfunction and adverse cardiovascular effects [128,129,130].

Several studies have observed accumulation of AGE in native and transplant CKD patients, and a strong body of evidence on the general theory of AGE pathophysiology supports its pivotal role in the initiation and progression of mechanisms underlying cardiovascular disease. However, few attempts have been made to investigate the association of AGE with cardiovascular risk post-kidney transplantation [99,131]. Through a mediating effect on up-regulation of inflammatory, oxidative stress and endothelial dysfunction biomarkers, a relative increase of AGE may be hypothesized to actively contribute to the intracellular signaling pathways that ultimately yield excess risk of premature cardiovascular death in KTR. It remains unknown whether a hypothetical association with risk of cardiovascular mortality is independent of estimates of kidney function and traditional cardiovascular risk factors such as body mass index, diabetes, blood pressure, and smoking status.

#### 3.1.3. Inflammation, Galectin-3, and Fibrosis

Inflammation is also referred to as a unifying mechanism of injury because—through a cornerstone signaling link with interstitial fibrosis and tubular atrophy—it may hold observations that connect hazards of several natures with structural damage and detrimental function of the kidney [12,13,14,15,132,133]. Of note, the concept that chronic rejection is responsible for all progressive long-term kidney graft failure has long ago been reformulated to a hypothesis of cumulative damage [12,13,14,15]. Thus, repeated insults of both immune and non-immune nature damage the graft by leading to interstitial fibrosis and tubular atrophy, which represents a final common pathway of injury with adverse functional consequences [13]. Galectin-3 is a β-galactoside-binding lectin with a postulated key mediating role on kidney tissue fibrosis [134,135,136,137,138]. In different models, it has been shown that whether a variety of insults incur on irreversible kidney fibrosis or not depends on the expression and secretion of galectin-3 [135,136,137,138]. In the general population, moreover, an increasing body of prospective evidence has related plasma galectin-3 with incident CKD [139,140,141]. Because galectin-3 is both a biomarker of systemic inflammation and kidney fibrosis, it may broaden our understanding and provide data to further support a unifying link between repeated inflammatory and pro-oxidant insults and increased risk of graft failure beyond the first-year post-kidney transplantation. Finally, it should be realized that the dependent role of galectin-3 on kidney fibrosis has been specifically shown in the particular post-kidney transplant setting in a murine model [138]. Within the clinical kidney transplantation field, however, a number of crucial questions remain unanswered. Especially with galectin-3, targeted pharmacological therapies are increasingly becoming available, and evidence of a hypothetical association between galectin-3 levels and risk of long-term graft survival may point towards novel interventional avenues to potentially decrease the long-standing burden of late graft failure.

### 3.2. Bone Disease and Vascular Calcification

**Figure 4:**
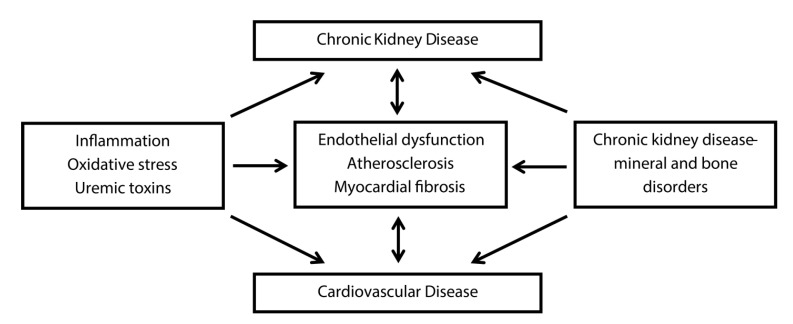
Cardiovascular disease in chronic kidney disease. This figure shows inflammation, oxidative stress, and uremic toxins on one side and chronic kidney disease-mineral and bone disorders on the other side of independent mechanisms linking chronic kidney and cardiovascular disease. Adapted from: “Cardiovascular disease in dialysis patients” by M. Cozzolino et al., 2019, Nephrol Dial Transplant, 33: iii28–34 [145].

Chronic kidney disease-mineral and bone disorders (CKD-MBD) is the clinical entity or syndrome that KDIGO (Kidney Disease: Improving Global Outcomes) more than a decade ago has coined to embody the disruption of the complex systems biology enclosed by the kidney, skeleton, and cardiovascular system [142]. In line with previous evidence, the results of a recent elegant study by Yilmaz et al. support the hypothesis that decline in cardiovascular risk post-kidney transplantation depends on partial resolution of inflammation but also on resolution of the CKD-MBD [143,144]. The findings of the aforementioned research group support the notion that beyond restoration of organ function post-kidney transplant, amelioration of inflammation and correction of CKD-MBD may attenuate excess cardiovascular disease through separate biological pathways. In agreement, Cozzolino et al. recently depicted inflammation and oxidative stress, on one hand, and CKD-MBD, on the other hand, as major mechanisms underlying a feedback loop that exacerbates cardiovascular disease in CKD patients (Figure 4) [145].

Within the context of CKD-MBD, vascular calcification—a currently established cardiovascular risk factor in KTR, as shown by previous studies of our group and others [146,147,148,149,150,151]—is linked with bone disease through inter-related pathophysiological mechanisms that comprise the bone-vascular axis hypothesis, which contributes to the exceedingly high cardiovascular risk in native CKD [152,153,154,155,156]. Post-kidney transplant bone disease is certainly a topic of epidemiological relevance due to its high prevalence and its association with fragility fractures and reduced mobility [157,158,159,160,161,162]. Previous studies remarked that existing research had failed to explore a hypothetical contributing role of post-kidney transplant bone disease to increased risk of vascular calcification in KTR [154,158,163]. Recent evidence, however, has come to support the existence of a bone-vascular axis post-kidney transplantation, providing data to evaluate its epidemiological relevance post-kidney transplant and pointing towards an otherwise overlooked therapeutic opportunity to at least partially decrease the markedly high cardiovascular burden post-kidney transplant [164].

It has also been proposed that mediators of inflammation (e.g., interleukin 6 and tumor necrosis factor) contribute to fibroblast growth factor (FGF)-23 elevation and that, in turn, FGF-23 increases cytokine production, thus linking systemic inflammation with dysregulated phosphate metabolism in a vicious cycle [165,166]. It has been proposed that inflammatory mediators function as drug targets to decrease the burden of FGF23-associated injury in various tissues, thus offering a novel therapeutic opportunity to decrease the burden of cardiovascular diseases including vascular calcification in kidney disease patients [165,167]. Nevertheless, even in CKD patients within normal range of serum phosphate levels, vascular calcification is often observed. Calciprotein particles are calcium-phosphate nanoparticles that increase with CKD progression, which have been associated with inflammatory responses, endothelial damage, vascular stiffness, and calcification [168]. Calciprotein particles may play a pathophysiological role in the link between chronic inflammation and vascular calcification. Further research is warranted to evaluate its contribution to overall cardiovascular burden in KTR and to develop novel pharmacological strategies targeting calciprotein particles to encourage protection against the risk of vascular calcification post-kidney transplantation [169].

### 3.3. Immunosuppressive Therapy and Traditional Risk Factors of Vascular Calcification

The contribution of several traditional risk factors of vascular calcification may be particularly relevant in the post-kidney transplantation setting due to the effect of maintenance immunosuppressive therapy on diabetes, dyslipidemia, and vitamin D metabolism [170]. Previous studies have shown that low vitamin D along with low vitamin K may synergistically associate with higher risk of hypertension [171] and thereby contribute to higher risk of vascular calcification [172]. In KTR, particularly, we have recently shown that combined vitamin D and K deficiency is highly prevalent and is associated with increased mortality and graft failure [173]. Further research is needed to investigate both the direct and indirect role of immunosuppressive drugs in the progression of vascular calcification. There may, however, be opposing effects, because it has been described that steroids and calcineurin inhibitors inhibit inducible nitric oxide and may thereby lead to progression of vascular calcification through endothelial dysfunction [170], while mycophenolate mofetil inhibits vascular smooth muscle cell proliferation and may be protective against vascular calcification [174,175]. Similarly, we recently reported that use of cyclosporine rather than tacrolimus correlated with prevalence of osteopenia, while osteopenia was associated with higher risk of vascular calcification after kidney transplantation [164]. Future studies are warranted to assess the association between immunosuppressive agents and risk of vascular calcification, which may provide new cardiovascular risk management opportunities post-kidney transplantation.

## 4. Conclusions

Further research on lifestyle-related factors including diet and exposure to toxic contaminants, as well as persisting mechanisms of disease post-kidney transplantation (i.e., inflammation and redox imbalance and vascular calcification) is needed as it may bring about powerful opportunities to improve long-term outcomes post-kidney transplantation. Reflection on these current research opportunities is warranted among the research and clinical kidney transplantation community. Forthcoming analyses of the data to be generated by the long-lasting Transplant Lines Prospective Cohort Study and Biobank of Solid Organ Transplant Recipients [176] may shed light on these questions.
